# Screening of Natural Compounds for CYP11A1 Stimulation Against Cell Renal Cell Carcinoma

**DOI:** 10.1186/s12575-023-00225-y

**Published:** 2023-11-30

**Authors:** Hien Thi My Ong, Eda Ates, Oh-Seung Kwon, Min-Jung Kang

**Affiliations:** 1 Center for Advanced Biomolecular Recognition, Korea Institute of Science and Technology, Seoul, 02792 Republic of Korea; 2 Division of Bio-Medical Science & Technology, KIST School, University of Science and Technology, Seoul, 02792 Republic of Korea; 3 Doping Control Center, Korea Institute of Science and Technology, Seoul, 02792 Republic of Korea

**Keywords:** CYP11A1-overexpressing kidney cancer cell model, Quantitative analysis of cholesterol and pregnenolone, LC–MS/MS, Autophagy, Ferroptosis

## Abstract

**Background:**

Renal cancer therapies are challenging owing to the extensive spreading of this cancer to other organs and its ability to pose resistance to current medications. Therefore, drugs targeting novel targets are urgently required to overcome these challenges. The cholesterol side-chain cleavage enzyme (CYP11A1) is closely associated with steroidogenesis, and its downregulation is linked to adrenal dysfunction and several types of carcinoma. We previously found that overexpression of CYP11A1 inhibited epithelial-mesenchymal transition and induced G2/M arrest in the kidney cancer Caki-1 cell line. In this context, natural compounds that exhibit potent CYP11A1 stimulation activity can be promising therpaeutic agents for kidney cancer.

**Methods:**

We screened a panel of 1374 natural compounds in a wound-healing assay using CYP11A1-transfected Caki-1 cells. Of these, 167 promising biologically active compounds that inhibited cancer cell migration by more than 75% were selected, and their half-maximal inhibitory concentrations (IC_50_) were determined. The IC_50_ of 159 compounds was determined and 38 compounds with IC_50_ values less than 50 µM were selected for further analysis. Steroid hormones (cholesterol and pregnenolone) levels in cells treated with the selected compounds were quantitated using LC–MS/MS to determine their effect on CYP11A1 activity. Western blotting for CYP11A1, autophagy signaling proteins, and ferroptosis regulators were performed to ivestigate the mechanisms underlying the action of the selected compounds.

**Results:**

We screened five promising natural lead compounds that inhibited cancer cell proliferation after three screening steps. The IC_50_ of these compounds was determined to be between 5.9 and 14.6 μM. These candidate compounds increased the expression of CYP11A1 and suppressed cholesterol levels while increasing pregnenolone levels, which is consistent with the activation of CYP11A1. Our results showed that CYP11A1 activation inhibited the migration of cancer cells, promoted ferroptosis, and triggered autophagy signaling.

**Conclusions:**

This study indicates that the CYP11A1-overexpressing Caki-1 cell line is useful for screening drugs against kidney cancer. The two selected compounds could be utilized as lead compounds for anticancer drug discovery, and specifically for the development of antirenal cancer medication.

**Supplementary Information:**

The online version contains supplementary material available at 10.1186/s12575-023-00225-y.

## Background

Kidney cancer generally occurs in kidney tissues and includes renal cell carcinoma (RCC), renal pelvis carcinoma, and Wilms tumor. With no apparent symptoms, diagnosis and treatment of RCC in early stages is difficult. Although surgical treatment is preferred, it is associated with a high risk of postoperative metastasis and recurrence [1]. The other treatment modalities, such as radiotherapy and chemotherapy, have their limitations of low sensitivity and side effects [2]. In recent years, immunotherapy has emerged as a promising therapeutic strategy; however, its application remains complicated owing to different tumor microenvironments. Blocking a single checkpoint can activate or suppress other immune modulators [3]. Dysregulation of steroid hormones also plays an important role in the early and late stages of renal cancer. Metabolism of cholesterol and biosynthesis of other lipids is directly associated with RCC through the accumulation of cholesterol, lipids, and glycogen [4]. The hereditary kidney cancer gene, *TRC8* is a key regulator of endogenous cholesterol degradation that suppresses RCC growth [5]. CYP11A1 catalyzes the first step in steroidogenesis by hydroxylating cholesterol to pregnenolone. Significant downregulation of CYP11A1 has been reported in kidney and five other cancers [6]. Pregnenolone and its derivatives exhibit significant cytotoxic activity against lung cancer [7], and are used as models for anticancer drugs [8]. Most Food and Drug Administration-approved drugs that are widely used in cancer therapeutics face the problem of resistance during the initial phase of drug treatment. Although cancer prevention is preferable over treatment, research on new anticancer drugs is still the primary concern considering drug resistance. It is particularly important to identify drugs that inhibit, interfere with, or reverse cancer proliferation. Cancer metastasis is a complex, multistep process, with the invasion of tumor into the extracellular matrix playing an important role in it [9]. Tumor invasion involves the following three steps: (i) attachment of tumor cells to matrix components, (ii) local degradation of the matrix by tumor cell-associated proteases, and (iii) migration of tumor cells into the region of the matrix modified by proteolysis. The inhibition of any of these steps can inhibit tumor invasion, leading to a reduction in tumor metastasis [10].

Natural compounds have long been of interest because of their anticancer functions and play a dominant role in the development of pharmaceuticals for cancer treatment. Many of these compounds were first discovered as anticancer drugs for RCC, and are currently used in clinical practice. Pembrolizumab, has been approved by the Food and Drug Administration of the USA (FDA) as a primary adjuvant therapy for patients with RCC [11]. Lenvatinib, an organic compound that acts as an inhibitor of vascular endothelial growth factor receptors, has been found to be effective in metastatic RCC [12]. Mitomycin C occurs naturally in *Streptomyces caespitosus* and has been used to treat renal tumors in the form of microcapsules [13]. Natural product research is an effective approach for discovering bioactive compounds with proven therapeutic efficiency and known mechanisms of action.

We recently reported that overexpression of CYP11A1 reverses the epithelial-mesenchymal transition by arresting the kidney cancer cells in G2/M phase [14]. Therefore, we designed a screening model using a CYP11A1-overexpressing kidney cancer cell line to identify lead compounds for the treatment of kidney cancer. First, a panel of 1374 natural compounds was tested for cancer inhibition in the CYP11A1-overexpressing Caki-1 cell line model using a wound healing assay. Second, the half-maximal inhibitory concentrations (IC_50_) of the compounds were measured to screen the ones effective against kidney cancer. Third, label-free quantitative analysis of the substrate (cholesterol) and product of CYP11A1 (pregnenolone) was performed to select the natural compounds that activated CYP11A1. Furthermore, the possible anticancer actions of the selected natural compounds were investigated.

## Methods

### Materials

CYP11A1 cDNA, cloned in a pCMV-SPORT5 vector (Clone ID: hMU004796), was obtained from Korea Gene Bank. Plasmid Midi Kit was purchased from Qiagen (CA, USA). Dulbecco's modified Eagle’s medium–high glucose (DMEM) was purchased from GenDEPOT (TX, USA). Fetal bovine serum (FBS) and 1% penicillin/streptomycin solution were purchased from GIBCO (MA, USA). Lipofectamine 3000 reagent was procured from Invitrogen (MA, USA). RIPA buffer, protease, and phosphatase inhibitor cocktail were obtained from Cell Signaling (MA, USA) and Pierce™ BCA Protein Assay Kit and Pierce™ Protein A/G Agarose was purchased from Thermo Scientific (MA, USA).

Cholesterol, pregenenolone, mitomycin C, and aminoglutethimide standards were purchased from Selleck Chemicals LLC (TX, USA). The natural products set consisting of 1374 compounds in 5 μL volumes in 96-well polypropylene microtiter plates at concentrations ranging from 3.7 to 14.7 mM was kindly provided by the Korea Chemical Bank.

Primary antibodies directed against CYP11A1 (#14,217), Beclin1 (#3495), and LC3A/B (#12,741), horseradish peroxidase (HRP)-conjugated anti-rabbit IgG (#7074), and Ferroptosis Antibody Sampler Kit (#29,650) were purchased from Cell Signaling (MA, USA). Antibody against GAPDH was purchased from GeneTex (CA, USA).

### Cell Culture and Transfection

Plasmid cDNA was isolated using the plasmid midi kit according to the manufacturer’s instructions. The concentration of CYP11A1 cDNA was measured using a NanoDrop spectrophotometer. Caki-1 cells were cultured in DMEM-high glucose containing 10% FBS and 1% penicillin/streptomycin solution. Culture plates were incubated at 37 °C with 5% CO_2_ in a humidified cell incubator. The cells were transiently transfected with CYP11A1 using Lipofectamine 3000 following the manufacturer’s protocol. All experiments were performed using cells in logarithmic growth phase.

For protein quantification, cells were collected in cold phosphate-buffered saline (PBS), washed twice with PBS, and then lysed in RIPA lysis buffer containing a protease and phosphatase inhibitor cocktail. The cell lysates were incubated at 4 °C for 20 min and vortexed every 7 min. After centrifugation of the sample for 20 min at 14,000 × *g* at 4 °C, the supernatant was collected. Protein concentration in the lysate was determined using the Pierce™ BCA Protein Assay Kit according to the manufacturer’s protocol.

### Wound-Healing Assay: 1^st^ Screening

All 1374 compounds were screened at a final concentration of 10 μM with CYP11A1-overexpressing Caki-1 cells. Caki-1 cells (4 × 10^3^) were cultured in 24-well plates at 37 °C with 5% CO_2_) until they reached 90% confluence. These cells were transfected with CYP11a1 plasmid DNA using Lipofectamine 3000 reagent for 24 h. The natural compounds were diluted to a final concentration at 10 μM and added to each well of the 24-well plate. Scratch wound-healing assay was performed by scratching across the confluent cell monolayer with a sterilized 200 μL pipette tip. Cell debris was removed by extensive washing with 1 × PBS and cells were allowed to migrate into the wound area for 24 h at 37 °C. Digital photographs were taken at 0 and 24 h after scratching. The ImageJ 1.53a software (National Institutes of Health, Bethesda, MD, USA) was used to measure the width of the wounds at three locations within each well. The percentage wound closure was quantified by dividing the width of healed wounds at 24 h with the initial width. Compounds that inhibited the migration of cancer cells by more than 75% were selected. The compounds that did not exhibit the wound-healing activity or killed 100% of cells were excluded from further analysis.

### Cell Viability Assay for IC_50_ Determination: 2^nd^ Screening

The viability of Caki-1 cells treated with 159 compounds was evaluated using an EZ-Cytox assay (DoGenBio, Seoul, South Korea). The cells were seeded into 96-well plates at a density of 1 × 10^4^ cells/well and cultured for 24 h. They were subsequently treated with various concentrations of each compound (0, 1.5625, 3.125, 6.25, 12.5, 25, 50, 100 μM) for 24 h. After treatment, EZ-Cytox solution (10 μL) was added to each well, and the cells were incubated for 1 h, protected from light. The absorbance was measured at 450 nm using a Bio-Rad microplate reader (CA, USA). Cell viability was calculated using the following formula: % cell viability = (OD of treatment − OD of blank)/(OD of control − OD of blank) × 100%. The assay was repeated three times. An IC_50_ lower than 50 μM was the selection criteria.

### Assay of Enzymatic Activity: 3^rd^ Screening

To test the effect of 38 selected compounds on CYP11A1 activity, we developed a quantitative analysis method for the steroid hormones, cholesterol and pregnenolone, using LC–MS/MS.

### LC–MS/MS procedure

To determine cholesterol and pregnenolone concentrations, culture media (CM) samples were collected and processed using the liquid–liquid extraction method. Ethyl acetate and CM samples were mixed at a ratio of 5:1 (v/v) by vortexing for 1 min and then shaken in a rotator for 30 min. The mixture was then centrifuged at 1000 × *g* for 10 min at 4 °C, which allowed the solvent layer to be separated. Samples were frozen at − 80 °C and supernatant was transferred into a new clean tube. The liquid extraction was repeated two times for maximum recovery. The pooled supernatants were evaporated under a nitrogen stream. Samples were stored at − 20 °C until analysis.

Steroid analysis was conducted using a UHPLC-MS/MS system combined with an LTQ Orbitrap Velos Pro. A CORTECS C18 column (90 Å, 2.7 µm, 2.1 mm × 50 mm) (Waters Corporation, MA, USA) was used for separation. The column oven was maintained at 40 °C, and the injection volume was 10 μL. Isopropanol or 0.1% formic acid in water was used as a mobile phase at a flow rate of 0.4 mL/min. The gradient elution and MS operating conditions are presented in Table 1. The mass spectrometer was operated in positive electrospray ionization mode with total ion monitoring or multi-reaction monitoring. The spray voltage of the Orbitrap mass spectrometer was + 3.9 kV and the collision energy was 35 eV.

**Table 1:** High performance liquid chromatography gradient and mass spectrometry operating condition

**HPLC gradient**					
**Time (min)**	**Mobile A (%)**		**Mobile B (%)**	**Flow rate (mL/min)**	
0	100		0	0.4	
1	75		25	0.4	
2	50		50	0.4	
6	0		100	0.4	
7.5	0		100	0.4	
8	100		0	0.4	
10	100		0	0.4	
**MS operating condition**					
Mode	ESI positive mode				
Scan type	Full scan or multi reaction monitoring				
**Compounds**	**MW**	**[M + H]**^**+**^	**Ion transition**	**Collision energy**	**Retention time (min)**
Cholesterol	386.65	369.35	369.35 → 243.15	35	6.67
Pregnenolone	316.48	299.26	299.26 → 281.23	35	4.49
Finastride (IS)	372.54	373.28	373.28 → 305.22	35	4.14

### Method Validation

Calibration curves were constructed using 0.05–25 µg/mL cholesterol and pregnenolone standards, with finasteride as an internal standard (10 ng/mL). Six different concentrations of the standards were added to the cell culture medium and extracted using the method described above. The recovery was determined by calculating the mean percentage of the extracted sample area divided by the standard area. To calculate the limit of detection (LOD) and limit of quantification (LOQ) of steroid hormones, five replicates of low concentrations of target analytes were measured, and the concentrations that yielded a signal-to-noise ratio (S/N) of 3 for LOD and 10 for LOQ were selected. The accuracy of the method was determined as the % ratio of the measured quality control sample concentrations calculated using the calibration curve to the theoretical concentrations (1 and 10 µg/mL). Precision was calculated as the coefficient of variation (CV%) for triplicate measurements. The accuracy and precision of the method were assessed using intra- and inter-day variations from three repeated analyses.

### Western Blot Analysis and Immunoprecipitation

Protein samples were separated using one-dimensional 12% Tris–glycine SDS-PAGE, and the proteins were transferred onto nitrocellulose membranes (Bio-Rad, CA, USA). The membrane was blocked by incubating in 5% skim milk in 1X TBS with 0.5% Tween 20 for 1 h and then washed with 1X TBST. The membranes were incubated overnight with primary antibodies diluted 1:1000 (v:v) in 5% BSA at 4 °C in the dark. After washing three times with TBST for 5 min each time, the membranes were incubated with horseradish peroxidase-conjugated secondary antibody (MA, USA) in 5% skim milk (1:5000 v:v diluted) at room temperature for 1 h. The blots were detected using a chemiluminescence SignalFire™ ECL Reagent from Cell Signaling Technology (MA, USA) with an Ez-Capture MG system ATTO (NY, USA). The relative intensities of the western blot bands were measured using the ImageJ software (MD, USA). The GAPDH antibody was used as a loading control.

Immunoprecipitation was performed using cell lysates with 500 µg of total protein and antibodies against CYP11A1. Cell lysate was incubated with 50 µL of Protein A/G agarose bead slurry for precleaning. The protein concentration was 1 mg/mL. The samples were mixed by rotation at 4 °C for 60 min, microcentrifuged for 10 min at 4 °C, and the supernatant was transferred to a fresh tube. The primary antibody (diluted 1:50, v:v) was added to 500 µg of precleaned cell lysates and the mixtures were incubated overnight with rotation at 4 °C. Thereafter, 50 µL of Protein A/G agarose bead slurry was added and the mixture was incubated with rotation at 4 °C for 1–3 h. The mixtures were microcentrifuged for 30 s at 4 °C. The pellet was washed five times with 500 µL of 1X cell lysis buffer (kept on ice between washes) and used for immunoblotting after SDS-PAGE.

### Lipid Reactive Oxygen Species Assay

Cells, cultured to an appropriate density (5 × 10^7^ cells), were collected and washed twice with ice-cold PBS. An EZ-Lipid Peroxidation (TBARS) Assay Kit (DoGenBio, Seoul, South Korea) was used to detect the malondialdehyde (MDA) levels, which reflect the level of lipid oxidation. The absorbance in this colorimetric assay was measured at 540 nm using a Bio-Rad microplate reader (CA, USA). The relative MDA levels were calculated according to the manufacturer’s protocol.

### Statistical Analysis

All data are presented as mean ± standard deviation (SD) of triplicate results. *p ≤ 0.05, **p ≤ 0.01, ***p ≤ 0.0001, and ****p ≤ 0.0001 were considered to indicate significant differences. One-way ANOVA followed by Dunnett’s multiple comparison test was performed using GraphPad Prism version 8.0.0 for Windows (GraphPad Software, CA, USA).

## Results

### Establishment of CYP11A1-Overexpressing Cell-Based Model for Anticancer Drug Screening

**Fig. 1:**
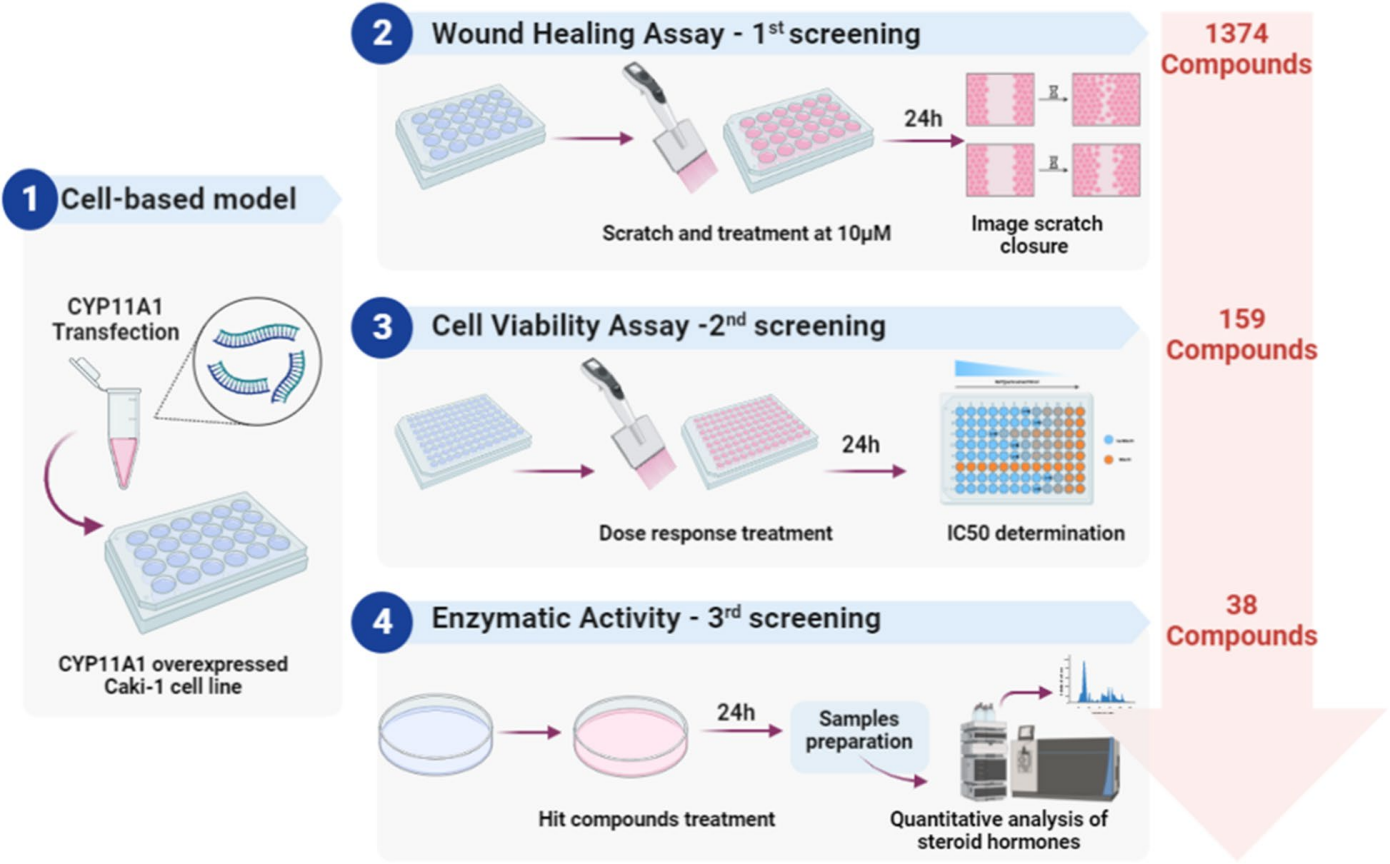
Schematic representation of targeting CYP11A1 in the cell-based assay established for screening of anticancer compounds

**Fig. 2:**
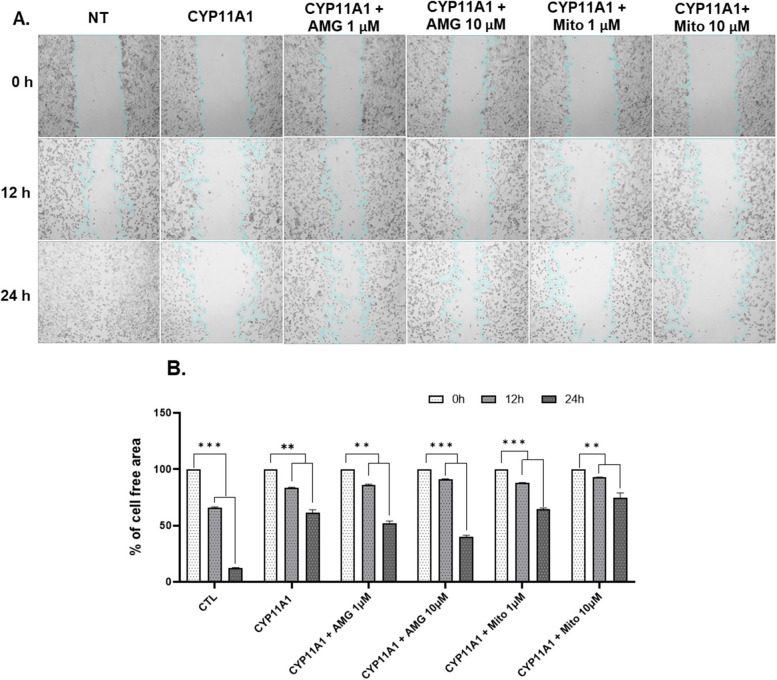
Wound-healing assay. **A** Caki-1 cells were transfected with CYP11A1 and then treated with 1 and 10 µM of aminoglutethimide (AMG) and mitomycin C (Mito). The wound area at 0 h was set at 100% and was subsequently measured at 12 an 24 h of treatment. Multiple images were collected and analyzed using the ImageJ software. **B** Histogram showing % of cell-free regions; the significance of differences in areas relative to the corresponding controls is shown (*n* ≥ 3). **p < 0.01, ***p < 0.001, and ****p < 0.0001

The overall procedure is illustrated in Fig. 1. We created a three-step method that was optimized for cell-based assays for screening a library of 1374 compounds to identify CYP11A1-activating compounds as anticancer drug candidates. We first established a CYP11A1-overexpressing cell model. To test the feasibility of employing this model, we used aminoglutethimide (AMG) as a negative control (inhibitor of CYP11A1) and mitomycin C (Mito) as a positive control (activator of CYP11A1). AMG blocks the enzymatic activity of CYP11A1 and prevents the conversion of cholesterol into pregnenolone in serum [15]. Mito is an FDA-approved chemotherapeutic agent for the treatment of bladder, gastric, and pancreatic cancer treatment [16], which activates the enzymatic activity of CYP11A1 [17]. To confirm the activity of these compounds in the CYP11A1-overexpressing Caki-1 cells, we performed a wound-healing assay with 1 and 10 µM of the positive and negative control compounds. As shown in Fig. 2, the percentage of cell-free area was less than 75% and 50% after 12 and 24 h of AMG treatment, respectively, whereas it was greater than 75% after Mito treatment. At a lower Mito concentration (1 µM), the area of cell-free regions after 12 and 24 h was smaller than that at 10 µM. These results indicate that the CYP11A1-overexpressing cells can be used in anticancer drug screening by measuring cancer cell progression.

Cell migration, for example, during metastasis, is closely involved in the progression of various cancers. Therefore, we used our screening system to determine the effect of 1374 natural compounds on CYP11A1 activity by evaluating cell migration in the wound-healing assay. The effects of CYP11A1 inhibitor and activator (10 µM) on the cell migration were assessed as negative and positive controls, respectively, in each assay. Mito significantly inhibited the migration of cancer cells by more than 75% whereas AMG only caused 30% wound closure compared with that in untreated CYP11A1-overexpressing Caki-1 cells (60% wound closure) (Table 2). After 24 h treatment with 10 µM of natural compounds, the migration of Caki-1 cells was markedly inhibited and correlated with their effects on CYP11A1-transfected Caki-1 cells. These data suggest that CYP11A1 may be an effective therapeutic target of anticancer drugs for blocking cancer cell growth.

**Table 2:** Anti-migration effects of 169 compounds on CYP11A1-overexpressing Caki-1 cells

		**Compound name (1st screening)**	**Tested concentration (µM)**	**%of free cell area at 0 h**	**%of free cell area at 24 h**	***p*-value**	**Compound name (2nd screening)**	**Tested concentration (µM)**	**IC50 (µM)**
	Caki-1 cell line		─	100	12.5	****		─	─
	CYP11A1 overexpressed Caki-1 cell line		─	100	60.6	****		─	
	CYP11A1 +	AMG	10	100	30.2	****		1,5,10,25,50,100	25.5 ± 1.4
	CYP11A1 +	Mito	10	100	75.4	****		1,5,10,25,50,100	18.43 ± 1.2
1	CYP11A1 +	NPS-D1-000030-B12	10	100	97.7	****	CPK-M1-014010-G04	1,5,10,25,50,100	112.3 ± 1.6
2	CYP11A1 +	NPS-D1-000042-D04	10	100	97.6	***	CPK-M1-014010-H09	1,5,10,25,50,100	53.0 ± 1.7
3	CYP11A1 +	NPS-D1-000030-A11	10	100	96.5	****	CPK-M1-014009-E10	1,5,10,25,50,100	183.8 ± 0.8
4	CYP11A1 +	NPS-D1-000036-C07	10	100	95.8	****	CPK-M1-014010-C04	1,5,10,25,50,100	107.5 ± 2.0
5	CYP11A1 +	NPS-D1-000029-F08	10	100	95.0	*	CPK-M1-014009-C12	1,5,10,25,50,100	141.4 ± 1.5
6	CYP11A1 +	NPS-D1-000042-D10	10	100	94.7	****	CPK-M1-014009-H09	1,5,10,25,50,100	109.4 ± 1.8
7	CYP11A1 +	NPS-D1-000028-A06	10	100	94.7	*	CPK-M1-014009-H10	1,5,10,25,50,100	68.7 ± 1.9
8	CYP11A1 +	NPS-D1-000029-F05	10	100	94.6	****	CPK-M1-014010-H03	1,5,10,25,50,100	62.0 ± 2.6
9	CYP11A1 +	NPS-D1-000029-C05	10	100	93.7	****	CPK-M1-014009-C07	1,5,10,25,50,100	82.1 ± 2.1
10	CYP11A1 +	NPS-D1-000028-A03	10	100	93.7	*	CPK-M1-014009-A06	1,5,10,25,50,100	111.6 ± 1.8
11	CYP11A1 +	NPS-D1-000032-D08	10	100	93.4	**	CPK-M1-014009-C10	1,5,10,25,50,100	39.0 ± 3.4
12	CYP11A1 +	NPS-D1-000030-D10	10	100	93.1	*	CPK-M1-014009-B07	1,5,10,25,50,100	22.7 ± 2.6
13	CYP11A1 +	NPS-D1-000031-G04	10	100	93.1	**	CPK-M1-014009-C09	1,5,10,25,50,100	126.3 ± 1.4
14	CYP11A1 +	NPS-D1-000029-D06	10	100	92.7	**	CPK-M1-014009-H03	1,5,10,25,50,100	50.5 ± 3.3
15	CYP11A1 +	NPS-D1-000028-F12	10	100	92.1	**	CPK-M1-014009-A10	1,5,10,25,50,100	208.7 ± 1.0
16	CYP11A1 +	NPS-D1-000030-D11	10	100	91.7	****	CPK-M1-014010-E07	1,5,10,25,50,100	132.6 ± 1.0
17	CYP11A1 +	NPS-D1-000031-G07	10	100	91.5	****	CPK-M1-014009-D09	1,5,10,25,50,100	130.3 ± 1.3
18	CYP11A1 +	NPS-D1-000043-C03	10	100	91.3	****	─	─	─
19	CYP11A1 +	NPS-D1-000029-E08	10	100	90.3	**	CPK-M1-014010-A03	1,5,10,25,50,100	95.1 ± 1.3
20	CYP11A1 +	NPS-D1-000035-E10	10	100	90.3	*	CPK-M1-014009-D08	1,5,10,25,50,100	90.7 ± 2.0
21	CYP11A1 +	NPS-D1-000043-B11	10	100	90.1	**	─	─	─
22	CYP11A1 +	NPS-D1-000040-F11	10	100	89.9	**	CPK-M1-014009-C03	1,5,10,25,50,100	31.7 ± 3.8
23	CYP11A1 +	NPS-D1-000029-B07	10	100	89.7	*	CPK-M1-014010-E06	1,5,10,25,50,100	95.4 ± 1.5
24	CYP11A1 +	NPS-D1-000030-A05	10	100	89.3	*	CPK-M1-014010-G07	1,5,10,25,50,100	166.9 ± 1.0
25	CYP11A1 +	NPS-D1-000039-G07	10	100	89.0	*	CPK-M1-014009-B09	1,5,10,25,50,100	43.7 ± 3.0
26	CYP11A1 +	NPS-D1-000027-D11	10	100	89.0	*	CPK-M1-014010-A10	1,5,10,25,50,100	195.4 ± 1.0
27	CYP11A1 +	NPS-D1-000030-E06	10	100	88.9	*	CPK-M1-014009-F06	1,5,10,25,50,100	104.5 ± 2.0
28	CYP11A1 +	NPS-D1-000029-D07	10	100	88.9	*	CPK-M1-014009-F03	1,5,10,25,50,100	32.1 ± 1.9
29	CYP11A1 +	NPS-D1-000037-H03	10	100	88.8	****	CPK-M1-014009-C05	1,5,10,25,50,100	124.4 ± 1.3
30	CYP11A1 +	NPS-D1-000027-E12	10	100	88.5	*	CPK-M1-014009-C04	1,5,10,25,50,100	37.6 ± 4.3
31	CYP11A1 +	NPS-D1-000029-A09	10	100	88.4	****	CPK-M1-014010-G03	1,5,10,25,50,100	34.8 ± 3.0
32	CYP11A1 +	NPS-D1-000030-E08	10	100	88.2	*	CPK-M1-014010-C10	1,5,10,25,50,100	282.4 ± 1.4
33	CYP11A1 +	NPS-D1-000027-H12	10	100	87.9	*	CPK-M1-014010-H07	1,5,10,25,50,100	82.0 ± 2.4
34	CYP11A1 +	NPS-D1-000028-G10	10	100	87.7	*	CPK-M1-014009-F12	1,5,10,25,50,100	90.0 ± 1.6
35	CYP11A1 +	NPS-D1-000036-B11	10	100	87.3	*	CPK-M1-014010-F06	1,5,10,25,50,100	136.0 ± 1.5
36	CYP11A1 +	NPS-D1-000028-G04	10	100	87.0	*	CPK-M1-014010-H06	1,5,10,25,50,100	55.9 ± 3.4
37	CYP11A1 +	NPS-D1-000029-H08	10	100	86.9	****	CPK-M1-014009-C08	1,5,10,25,50,100	152.3 ± 1.3
38	CYP11A1 +	NPS-D1-000029-A06	10	100	86.7	*	CPK-M1-014009-D12	1,5,10,25,50,100	93.2 ± 2.3
39	CYP11A1 +	NPS-D1-000028-H05	10	100	86.5	****	CPK-M1-014009-G10	1,5,10,25,50,100	93.1 ± 2.6
40	CYP11A1 +	NPS-D1-000028-C03	10	100	86.3	*	CPK-M1-014010-F10	1,5,10,25,50,100	443.7 ± 4.2
41	CYP11A1 +	NPS-D1-000029-D05	10	100	86.3	****	CPK-M1-014010-A11	1,5,10,25,50,100	169.8 ± 1.0
42	CYP11A1 +	NPS-D1-000036-G08	10	100	86.0	****	CPK-M1-014010-G11	1,5,10,25,50,100	41.2 ± 3.0
43	CYP11A1 +	NPS-D1-000041-A10	10	100	85.9	*	CPK-M1-014009-D05	1,5,10,25,50,100	50.5 ± 2.0
44	CYP11A1 +	NPS-D1-000041-A11	10	100	85.8	*	CPK-M1-014009-H06	1,5,10,25,50,100	52.8 ± 3.8
45	CYP11A1 +	NPS-D1-000029-E09	10	100	85.8	*	CPK-M1-014010-A09	1,5,10,25,50,100	73.7 ± 3.0
46	CYP11A1 +	NPS-D1-000040-C10	10	100	85.7	*	CPK-M1-014010-E08	1,5,10,25,50,100	64.8 ± 1.8
47	CYP11A1 +	NPS-D1-000028-B08	10	100	85.6	*	CPK-M1-014010-A07	1,5,10,25,50,100	14.5 ± 3.9
48	CYP11A1 +	NPS-D1-000028-E12	10	100	85.6	**	CPK-M1-014009-B06	1,5,10,25,50,100	59.5 ± 4.3
49	CYP11A1 +	NPS-D1-000028-G11	10	100	85.1	*	CPK-M1-014010-B11	1,5,10,25,50,100	14.6 ± 3.8
50	CYP11A1 +	NPS-D1-000042-F04	10	100	85.0	****	CPK-M1-014010-F08	1,5,10,25,50,100	57.2 ± 3.0
51	CYP11A1 +	NPS-D1-000028-C07	10	100	84.9	****	CPK-M1-014009-G09	1,5,10,25,50,100	124.0 ± 1.8
52	CYP11A1 +	NPS-D1-000029-C07	10	100	84.8	*	CPK-M1-014009-E12	1,5,10,25,50,100	89.1 ± 1.2
53	CYP11A1 +	NPS-D1-000042-D09	10	100	84.8	*	CPK-M1-014010-E09	1,5,10,25,50,100	60.1 ± 2.2
54	CYP11A1 +	NPS-D1-000029-G05	10	100	84.7	*	CPK-M1-014010-D07	1,5,10,25,50,100	33.7 ± 3.0
55	CYP11A1 +	NPS-D1-000037-E04	10	100	84.5	*	CPK-M1-014009-D04	1,5,10,25,50,100	43.8 ± 4.1
56	CYP11A1 +	NPS-D1-000029-G07	10	100	84.4	****	CPK-M1-014009-D03	1,5,10,25,50,100	37.1 ± 3.9
57	CYP11A1 +	NPS-D1-000027-A11	10	100	84.4	**	CPK-M1-014010-F11	1,5,10,25,50,100	41.8 ± 4.2
58	CYP11A1 +	NPS-D1-000028-A04	10	100	84.3	****	CPK-M1-014009-G12	1,5,10,25,50,100	74.2 ± 1.5
59	CYP11A1 +	NPS-D1-000030-F05	10	100	84.1	****	CPK-M1-014009-G06	1,5,10,25,50,100	136.2 ± 1.6
60	CYP11A1 +	NPS-D1-000039-D07	10	100	84.0	****	CPK-M1-014010-H04	1,5,10,25,50,100	109.6 ± 2.0
61	CYP11A1 +	NPS-D1-000033-E07	10	100	84.0	**	CPK-M1-014009-C06	1,5,10,25,50,100	61.3 ± 3.5
62	CYP11A1 +	NPS-D1-000028-D03	10	100	84.0	*	CPK-M1-014010-E12	1,5,10,25,50,100	38.9 ± 2.9
63	CYP11A1 +	NPS-D1-000043-A10	10	100	83.9	****	CPK-M1-014010-G05	1,5,10,25,50,100	144.3 ± 1.2
64	CYP11A1 +	NPS-D1-000030-D06	10	100	83.8	*	CPK-M1-014010-E11	1,5,10,25,50,100	36.3 ± 2.1
65	CYP11A1 +	NPS-D1-000043-G05	10	100	83.8	*	─	─	─
66	CYP11A1 +	NPS-D1-000035-D08	10	100	83.8	****	CPK-M1-014009-E05	1,5,10,25,50,100	60.0 ± 1.7
67	CYP11A1 +	NPS-D1-000028-G12	10	100	83.5	*	CPK-M1-014010-C08	1,5,10,25,50,100	25.4 ± 2.0
68	CYP11A1 +	NPS-D1-000034-G08	10	100	83.5	*	CPK-M1-014009-F05	1,5,10,25,50,100	38.0 ± 2.8
69	CYP11A1 +	NPS-D1-000027-E11	10	100	83.0	**	CPK-M1-014010-E03	1,5,10,25,50,100	108.8 ± 2.1
70	CYP11A1 +	NPS-D1-000028-D12	10	100	82.8	*	CPK-M1-014009-F11	1,5,10,25,50,100	101.4 ± 2.0
71	CYP11A1 +	NPS-D1-000036-H07	10	100	82.7	****	CPK-M1-014010-F09	1,5,10,25,50,100	61.8 ± 2.5
72	CYP11A1 +	NPS-D1-000031-E04	10	100	82.7	**	CPK-M1-014010-C05	1,5,10,25,50,100	62.0 ± 1.8
73	CYP11A1 +	NPS-D1-000031-E06	10	100	82.6	****	CPK-M1-014009-D11	1,5,10,25,50,100	121.0 ± 1.9
74	CYP11A1 +	NPS-D1-000028-D05	10	100	82.4	**	CPK-M1-014009-C11	1,5,10,25,50,100	111.3 ± 1.9
75	CYP11A1 +	NPS-D1-000035-E05	10	100	82.2	*	CPK-M1-014010-B04	1,5,10,25,50,100	83.2 ± 2.1
76	CYP11A1 +	NPS-D1-000029-C08	10	100	82.2	*	CPK-M1-014009-G03	1,5,10,25,50,100	44.6 ± 3.7
77	CYP11A1 +	NPS-D1-000036-F03	10	100	82.1	****	CPK-M1-014010-C06	1,5,10,25,50,100	44.5 ± 3.8
78	CYP11A1 +	NPS-D1-000029-G08	10	100	82.0	**	CPK-M1-014010-G06	1,5,10,25,50,100	46.2 ± 2.7
79	CYP11A1 +	NPS-D1-000027-C12	10	100	82.0	**	CPK-M1-014009-F09	1,5,10,25,50,100	70.1 ± 2.8
80	CYP11A1 +	NPS-D1-000043-D06	10	100	81.9	****	─	─	─
81	CYP11A1 +	NPS-D1-000037-B04	10	100	81.9	****	CPK-M1-014010-D08	1,5,10,25,50,100	27.6 ± 3.4
82	CYP11A1 +	NPS-D1-000036-F06	10	100	81.8	**	CPK-M1-014009-F04	1,5,10,25,50,100	73.7 ± 2.1
83	CYP11A1 +	NPS-D1-000041-D12	10	100	81.7	**	CPK-M1-014009-H05	1,5,10,25,50,100	77.2 ± 2.1
84	CYP11A1 +	NPS-D1-000027-G11	10	100	81.7	*	CPK-M1-014009-G11	1,5,10,25,50,100	90.6 ± 1.9
85	CYP11A1 +	NPS-D1-000039-H03	10	100	81.6	*	CPK-M1-014009-B08	1,5,10,25,50,100	109.8 ± 1.7
86	CYP11A1 +	NPS-D1-000029-G04	10	100	81.5	*	CPK-M1-014009-E11	1,5,10,25,50,100	155.4 ± 1.5
87	CYP11A1 +	NPS-D1-000035-B09	10	100	81.5	**	CPK-M1-014009-D06	1,5,10,25,50,100	40.5 ± 3.6
88	CYP11A1 +	NPS-D1-000029-A07	10	100	81.4	**	CPK-M1-014009-B04	1,5,10,25,50,100	62.0 ± 1.4
89	CYP11A1 +	NPS-D1-000039-D04	10	100	81.2	**	CPK-M1-014010-F03	1,5,10,25,50,100	24.6 ± 1.8
90	CYP11A1 +	NPS-D1-000036-B05	10	100	81.0	***	CPK-M1-014010-D04	1,5,10,25,50,100	88.5 ± 2.0
91	CYP11A1 +	NPS-D1-000030-E09	10	100	80.9	*	CPK-M1-014010-C03	1,5,10,25,50,100	93.5 ± 1.7
92	CYP11A1 +	NPS-D1-000036-H06	10	100	80.8	****	CPK-M1-014009-G07	1,5,10,25,50,100	105.9 ± 1.7
93	CYP11A1 +	NPS-D1-000035-H09	10	100	80.8	*	CPK-M1-014010-B09	1,5,10,25,50,100	289.5 ± 0.8
94	CYP11A1 +	NPS-D1-000036-H05	10	100	80.7	****	CPK-M1-014010-D09	1,5,10,25,50,100	42.4 ± 3.3
95	CYP11A1 +	NPS-D1-000041-G10	10	100	80.6	*	CPK-M1-014010-E10	1,5,10,25,50,100	5.9 ± 2.1
96	CYP11A1 +	NPS-D1-000029-A05	10	100	80.5	****	CPK-M1-014009-A07	1,5,10,25,50,100	26.3 ± 3.1
97	CYP11A1 +	NPS-D1-000035-C10	10	100	80.3	****	CPK-M1-014009-H12	1,5,10,25,50,100	106.1 ± 1.5
98	CYP11A1 +	NPS-D1-000027-B11	10	100	80.2	*	CPK-M1-014010-D12	1,5,10,25,50,100	55.7 ± 2.6
99	CYP11A1 +	NPS-D1-000032-B04	10	100	79.9	**	CPK-M1-014009-A09	1,5,10,25,50,100	24.2 ± 1.8
100	CYP11A1 +	NPS-D1-000035-A09	10	100	79.8	**	CPK-M1-014010-B05	1,5,10,25,50,100	54.4 ± 2.1
101	CYP11A1 +	NPS-D1-000036-B06	10	100	79.7	**	CPK-M1-014010-G09	1,5,10,25,50,100	55.0 ± 2.3
102	CYP11A1 +	NPS-D1-000028-H11	10	100	79.7	*	CPK-M1-014009-B11	1,5,10,25,50,100	121.5 ± 1.7
103	CYP11A1 +	NPS-D1-000043-H09	10	100	79.7	*	─	─	─
104	CYP11A1 +	NPS-D1-000030-E05	10	100	79.6	****	CPK-M1-014009-A05	1,5,10,25,50,100	175.8 ± 1.2
105	CYP11A1 +	NPS-D1-000029-D08	10	100	79.6	*	CPK-M1-014010-F04	1,5,10,25,50,100	115.8 ± 1.8
106	CYP11A1 +	NPS-D1-000043-H03	10	100	79.5	*	─	─	─
107	CYP11A1 +	NPS-D1-000034-E09	10	100	79.5	**	CPK-M1-014009-A04	1,5,10,25,50,100	41.4 ± 2.6
108	CYP11A1 +	NPS-D1-000029-F09	10	100	79.4	****	CPK-M1-014009-B12	1,5,10,25,50,100	112.8 ± 2.0
109	CYP11A1 +	NPS-D1-000035-G03	10	100	79.1	*	CPK-M1-014010-H11	1,5,10,25,50,100	69.1 ± 1.5
110	CYP11A1 +	NPS-D1-000041-C12	10	100	79.1	*	CPK-M1-014009-F08	1,5,10,25,50,100	124.4 ± 1.5
111	CYP11A1 +	NPS-D1-000033-D07	10	100	79.0	****	CPK-M1-014009-E09	1,5,10,25,50,100	76.8 ± 2.3
112	CYP11A1 +	NPS-D1-000042-A03	10	100	78.9	**	CPK-M1-014010-F07	1,5,10,25,50,100	97.6 ± 2.0
113	CYP11A1 +	NPS-D1-000040-B08	10	100	78.9	***	CPK-M1-014010-D10	1,5,10,25,50,100	85.5 ± 3.3
114	CYP11A1 +	NPS-D1-000029-E07	10	100	78.9	**	CPK-M1-014009-G04	1,5,10,25,50,100	62.9 ± 3.8
115	CYP11A1 +	NPS-D1-000040-G11	10	100	78.8	***	CPK-M1-014010-E04	1,5,10,25,50,100	65.0 ± 2.4
116	CYP11A1 +	NPS-D1-000036-B09	10	100	78.5	****	CPK-M1-014010-A12	1,5,10,25,50,100	143.9 ± 1.5
117	CYP11A1 +	NPS-D1-000030-B09	10	100	78.5	*	CPK-M1-014009-E07	1,5,10,25,50,100	19.8 ± 2.0
118	CYP11A1 +	NPS-D1-000031-E07	10	100	78.5	****	CPK-M1-014010-B03	1,5,10,25,50,100	120.2 ± 1.4
119	CYP11A1 +	NPS-D1-000030-F12	10	100	78.4	****	CPK-M1-014010-D06	1,5,10,25,50,100	200.2 ± 0.9
120	CYP11A1 +	NPS-D1-000027-E06	10	100	78.3	****	CPK-M1-014010-B12	1,5,10,25,50,100	58.6 ± 3.5
121	CYP11A1 +	NPS-D1-000028-D04	10	100	78.1	*	CPK-M1-014009-D10	1,5,10,25,50,100	121.8 ± 1.9
122	CYP11A1 +	NPS-D1-000028-C04	10	100	78.0	****	CPK-M1-014010-C11	1,5,10,25,50,100	78.9 ± 1.4
123	CYP11A1 +	NPS-D1-000027-D10	10	100	77.9	**	CPK-M1-014010-D11	1,5,10,25,50,100	35.6 ± 3.3
124	CYP11A1 +	NPS-D1-000036-H11	10	100	77.8	***	CPK-M1-014009-A08	1,5,10,25,50,100	108.1 ± 2.0
125	CYP11A1 +	NPS-D1-000028-B04	10	100	77.7	****	CPK-M1-014010-G10	1,5,10,25,50,100	193.4 ± 3.0
126	CYP11A1 +	NPS-D1-000028-B12	10	100	77.6	****	CPK-M1-014009-E06	1,5,10,25,50,100	51.4 ± 2.9
127	CYP11A1 +	NPS-D1-000028-H03	10	100	77.6	***	CPK-M1-014010-A08	1,5,10,25,50,100	52.2 ± 1.7
128	CYP11A1 +	NPS-D1-000030-C11	10	100	77.5	**	CPK-M1-014009-H11	1,5,10,25,50,100	101.7 ± 1.4
129	CYP11A1 +	NPS-D1-000041-G08	10	100	77.4	**	CPK-M1-014010-H08	1,5,10,25,50,100	13.6 ± 2.1
130	CYP11A1 +	NPS-D1-000029-E05	10	100	77.3	****	CPK-M1-014010-B06	1,5,10,25,50,100	145.9 ± 1.6
131	CYP11A1 +	NPS-D1-000028-F05	10	100	77.2	*	CPK-M1-014009-F10	1,5,10,25,50,100	69.0 ± 1.5
132	CYP11A1 +	NPS-D1-000030-F09	10	100	77.2	**	CPK-M1-014009-H08	1,5,10,25,50,100	69.2 ± 1.5
133	CYP11A1 +	NPS-D1-000027-D12	10	100	77.0	*	CPK-M1-014009-G08	1,5,10,25,50,100	86.0 ± 2.2
134	CYP11A1 +	NPS-D1-000028-G05	10	100	76.9	*	CPK-M1-014010-A05	1,5,10,25,50,100	54.7 ± 2.5
135	CYP11A1 +	NPS-D1-000035-F09	10	100	76.8	****	CPK-M1-014010-B10	1,5,10,25,50,100	246.3 ± 3.8
136	CYP11A1 +	NPS-D1-000034-D07	10	100	76.7	**	CPK-M1-014010-B08	1,5,10,25,50,100	37.5 ± 2.8
137	CYP11A1 +	NPS-D1-000036-E08	10	100	76.7	*	CPK-M1-014009-G05	1,5,10,25,50,100	166.6 ± 1.4
138	CYP11A1 +	NPS-D1-000039-B07	10	100	76.5	**	CPK-M1-014010-D05	1,5,10,25,50,100	90.8 ± 0.8
139	CYP11A1 +	NPS-D1-000028-E10	10	100	76.5	****	CPK-M1-014010-H10	1,5,10,25,50,100	117.4 ± 1.5
140	CYP11A1 +	NPS-D1-000043-F08	10	100	76.5	****	─	─	─
141	CYP11A1 +	NPS-D1-000038-D11	10	100	76.5	**	CPK-M1-014009-H04	1,5,10,25,50,100	51.1 ± 3.8
142	CYP11A1 +	NPS-D1-000028-H08	10	100	76.4	*	CPK-M1-014009-B05	1,5,10,25,50,100	137.2 ± 1.6
143	CYP11A1 +	NPS-D1-000041-G07	10	100	76.2	****	CPK-M1-014010-C07	1,5,10,25,50,100	67.2 ± 1.8
144	CYP11A1 +	NPS-D1-000036-H03	10	100	76.2	**	CPK-M1-014010-F12	1,5,10,25,50,100	64.4 ± 1.6
145	CYP11A1 +	NPS-D1-000032-C03	10	100	76.2	**	CPK-M1-014009-B10	1,5,10,25,50,100	95.1 ± 1.5
146	CYP11A1 +	NPS-D1-000035-F10	10	100	76.2	**	CPK-M1-014009-E04	1,5,10,25,50,100	70.2 ± 1.9
147	CYP11A1 +	NPS-D1-000029-B08	10	100	76.2	****	CPK-M1-014009-H07	1,5,10,25,50,100	39.9 ± 2.6
148	CYP11A1 +	NPS-D1-000040-C11	10	100	76.0	*	CPK-M1-014010-E05	1,5,10,25,50,100	84.2 ± 1.5
149	CYP11A1 +	NPS-D1-000036-F07	10	100	75.9	***	CPK-M1-014010-A04	1,5,10,25,50,100	14.5 ± 1.0
150	CYP11A1 +	NPS-D1-000035-D11	10	100	75.9	*	CPK-M1-014010-C09	1,5,10,25,50,100	192.3 ± 1.1
151	CYP11A1 +	NPS-D1-000036-B12	10	100	75.8	*	CPK-M1-014009-A11	1,5,10,25,50,100	89.2 ± 2.3
152	CYP11A1 +	NPS-D1-000035-C12	10	100	75.6	****	CPK-M1-014010-D03	1,5,10,25,50,100	57.5 ± 2.0
153	CYP11A1 +	NPS-D1-000041-E11	10	100	75.6	****	CPK-M1-014009-E08	1,5,10,25,50,100	73.4 ± 2.4
154	CYP11A1 +	NPS-D1-000031-F04	10	100	75.6	*	CPK-M1-014010-H05	1,5,10,25,50,100	96.2 ± 1.7
155	CYP11A1 +	NPS-D1-000040-C08	10	100	75.6	**	CPK-M1-014010-A06	1,5,10,25,50,100	76.4 ± 2.5
156	CYP11A1 +	NPS-D1-000036-C05	10	100	75.4	**	CPK-M1-014009-A03	1,5,10,25,50,100	46.4 ± 2.0
157	CYP11A1 +	NPS-D1-000034-E10	10	100	75.3	****	CPK-M1-014009-A12	1,5,10,25,50,100	28.7 ± 3.6
158	CYP11A1 +	NPS-D1-000036-H04	10	100	75.3	*	CPK-M1-014010-F05	1,5,10,25,50,100	22.7 ± 2.4
159	CYP11A1 +	NPS-D1-000028-B10	10	100	75.3	*	CPK-M1-014010-G08	1,5,10,25,50,100	59.3 ± 1.5
160	CYP11A1 +	NPS-D1-000044-F04	10	100	75.2	****	─	─	─
161	CYP11A1 +	NPS-D1-000040-E10	10	100	75.2	**	CPK-M1-014010-B07	1,5,10,25,50,100	83.2 ± 2.1
162	CYP11A1 +	NPS-D1-000031-B04	10	100	75.2	****	CPK-M1-014009-D07	1,5,10,25,50,100	81.9 ± 1.8
163	CYP11A1 +	NPS-D1-000028-F04	10	100	75.1	*	CPK-M1-014010-C12	1,5,10,25,50,100	193.3 ± 0.9
164	CYP11A1 +	NPS-D1-000029-B05	10	100	75.1	****	CPK-M1-014009-E03	1,5,10,25,50,100	39.5 ± 2.5
165	CYP11A1 +	NPS-D1-000028-F03	10	100	75.0	**	CPK-M1-014009-F07	1,5,10,25,50,100	77.3 ± 2.0
166	CYP11A1 +	NPS-D1-000027-B10	10	100	75.0	**	CPK-M1-014010-G12	1,5,10,25,50,100	70.8 ± 1.6
167	CYP11A1 +	NPS-D1-000038-A12	10	100	75.0	****	CPK-M1-014009-B03	1,5,10,25,50,100	48.4 ± 1.9

The area of the scratched portion at time 0 was set as 100%; *p < 0.5, **p < 0.01, ***p < 0.001, and ****p < 0.0001

*ND*  Not determined because of detachment of cells from the surface

The results of the initial screening are summarized in Additional file 1. Among 1374 compounds, 167 inhibited the cell migration rate by ≥ 75%, whereas 406 and 317 compounds inhibited it by ≥ 50% and ≤ 50%, respectively. The effect of 484 compounds could not be determined because the cells detached from the surface after 24 h of treatment.

### Determination of IC_50_ of selected compounds

**Fig. 3:**
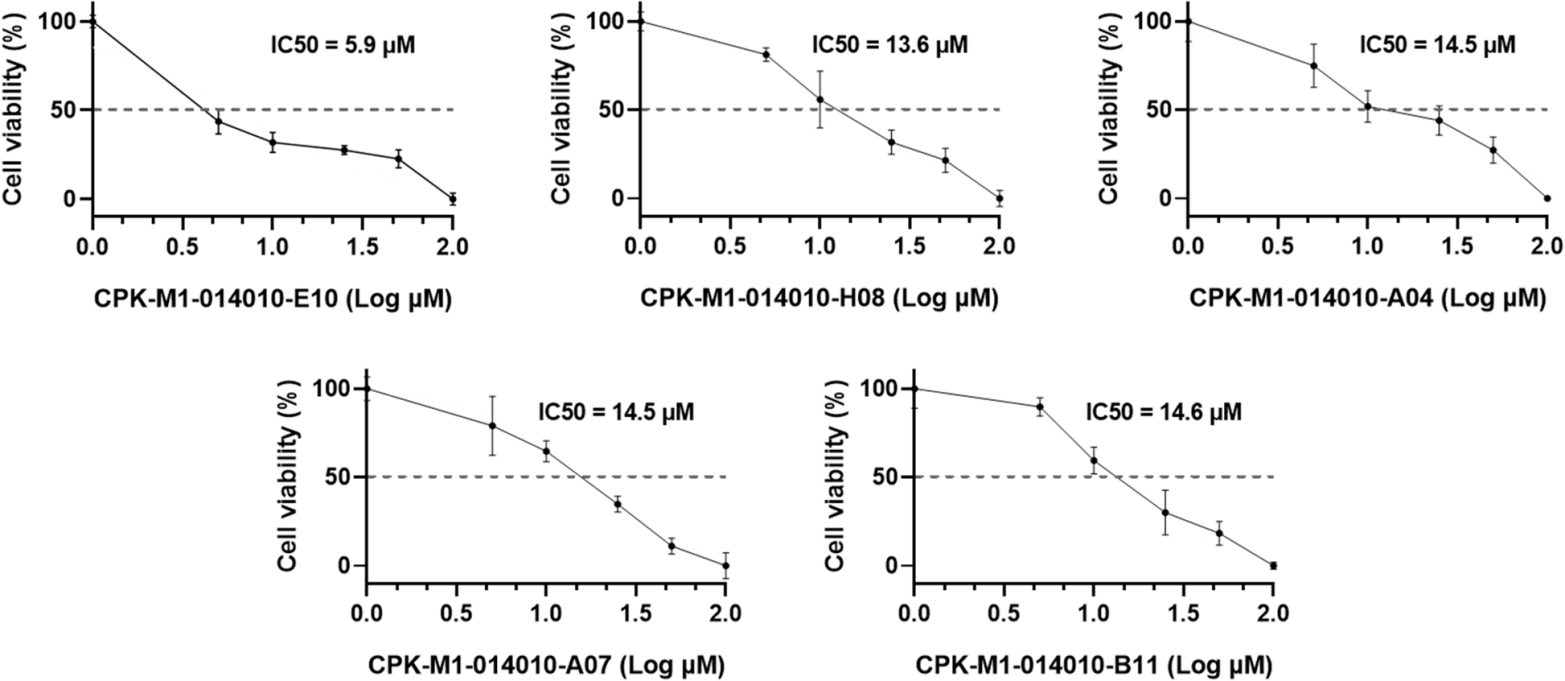
IC_50_ of natural compounds for CYP11A1-transfected Caki-1 cells. Cells were treated with different concentrations of the compounds and their viability was determined using the EZ-Cytox assay. The IC_50_ values for five selected compounds are shown

In the 2^nd^ screening, we further analyzed 159 natural compounds selected in the 1^st^ screening (of the 167 compounds, 8 were excluded because of low purity) by determining their IC_50_ values, which were calculated using calibration curves for dose-dependent percentage survival at seven concentrations. The IC_50_ values ranged from 5.9 μM to 443.7 μM. We selected 38 compounds with IC_50_ values less than 50 μM as potential candidates for assessing their anticancer effects on kidney cancer (Table 3). High IC_50_ values indicate negligible toxicity and low potential for cancer treatment. Five compounds with the highest IC_50_ values were CPK-M1-014010-E10, CPK-M1-014010-H08, CPK-M1-014010-A04, CPK-M1-014010-A07, and CPK-M1-014010-B11 (Fig. 3). The dose–response curves of these compounds correlated with the inhibition of cancer cell migration.

**Table 3:**
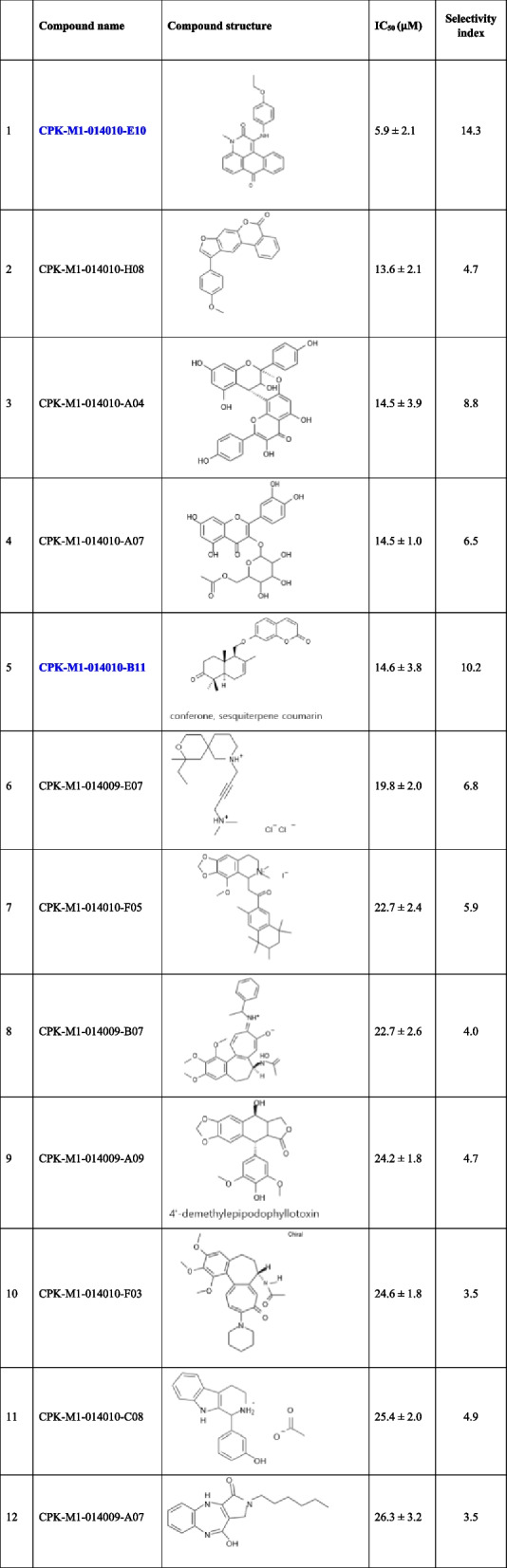

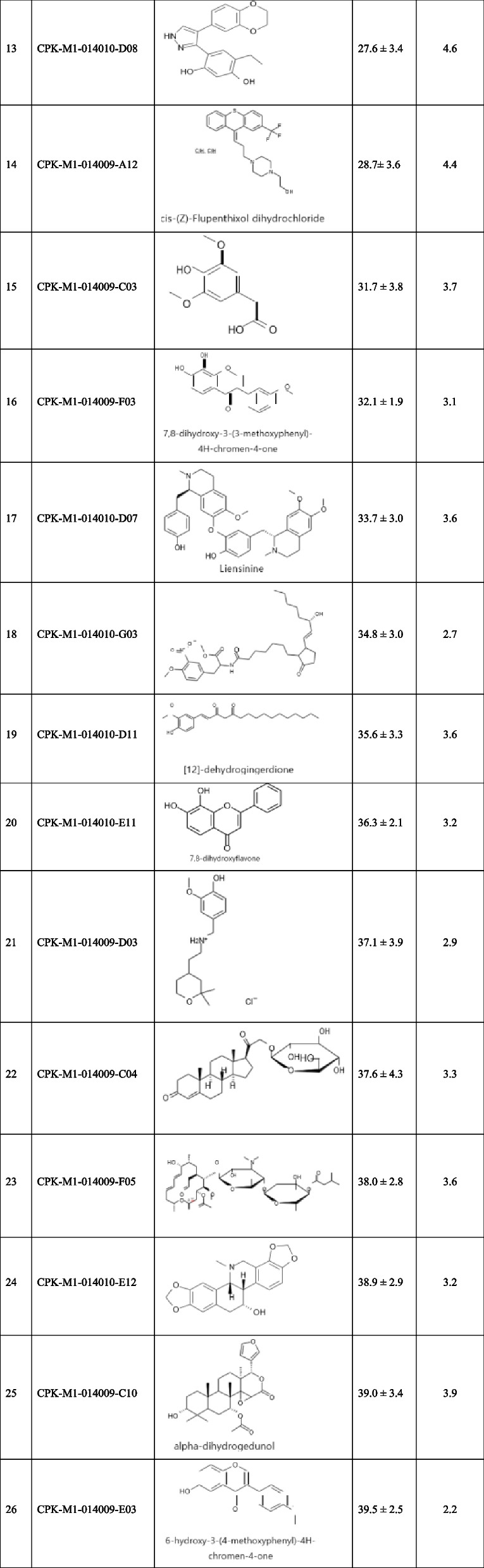

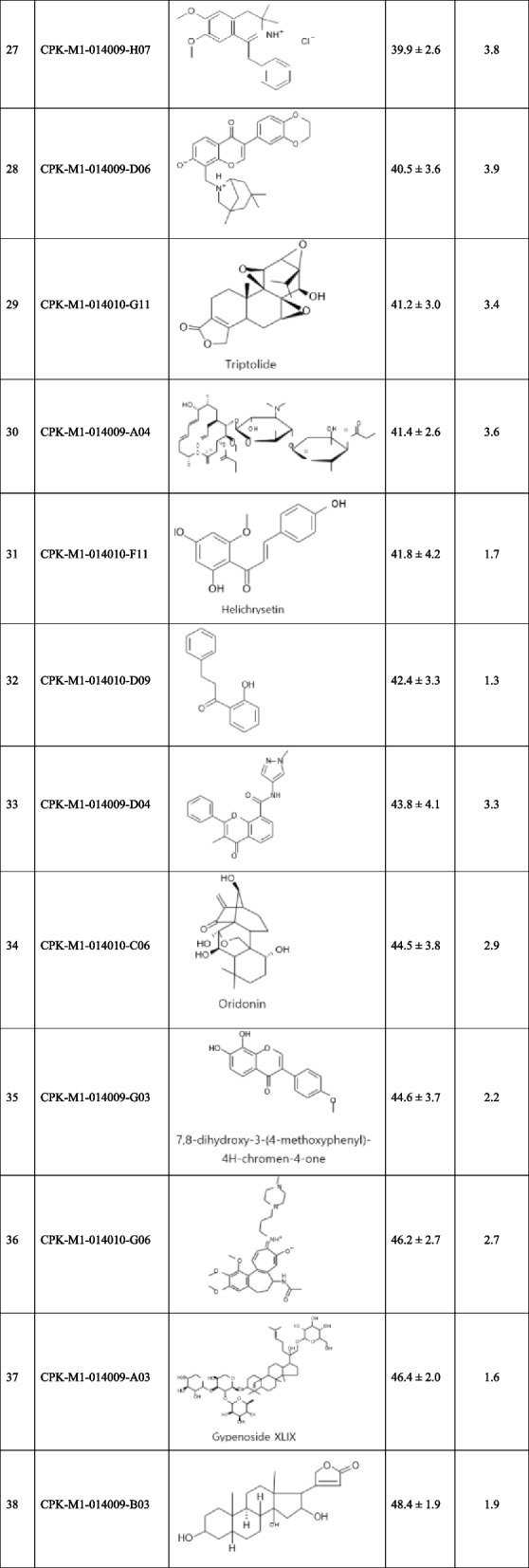
Half-maximal inhibitory concentration (IC_50_) and chemical structures of 38 selected compounds

### Validation of Method for Steroid Analysis

The development of the steroid analysis method started with the optimization of MS/MS parameters for target steroid analytes by injecting a standard solution (1 mg/mL in isopropanol containing 0.1% formic acid) directly into the mass spectrometer. Acetonitrile (ACN) and methanol (MEOH) produced a higher background than isopropanol; therefore, isopropanol was selected as the organic mobile phase. The chromatographic separation and MS/MS spectra of cholesterol, pregnenolone, and the internal standard finasteride are shown in Additional file 2. The method developed by us, with a total run time of 10 min, is fast and favorable for screening of compounds in large-scale studies.

The calibration curves were prepared by spiking 0.05–25 μg/mL of the standards in media and showed a linearity of 0.9995 and 0.9999 for pregnenolone and cholesterol, respectively (Table 4). The sensitivity of the method was determined as the LOD and LOQ. LODs ranged from 0.001 to 0.0014 ng/mL. The LOQs were 0.03 ng/mL for pregnenolone and 0.004 ng/mL for cholesterol. The average recoveries of cholesterol and pregnenolone were 78% and 80.9%, respectively. The recoveries were determined at different concentrations (0.1, 1, 10 μg/mL). The accuracy was in the range of 89.5–104%, and the relative standard deviation (SD) of triplicate measurements was 1.9–11.4%. The intraday CV for cholesterol was 2% and the interday CV was 11.3%. The intraday CV for pregnenolone was 3–3.9% and the interday CV was 3.9–4.2%. These results suggested acceptable precision (CVs < 20%) for all analyses and indicate that the method is feasible for quantitative analysis.

**Table 4:** Parameters for validation of the method established for quantittaion of target steroid hormones

	**LOD (ng/mL)**	**LOQ (ng/mL)**	**Calibration range (µg/mL)**	**Linearity**	**Concentration (µg/mL)**	**Inter day (*n* = 3)**		**Intra day (*n* = 3)**	
						**Accuracy** ** ± SD (%)**	**Precision (CV%)**	**Accuracy** ** ± SD (%)**	**Precision (CV%)**
Cholesterol	0.0014	0.004	0.05—25	0.9999	1	101.2 ± 11.4	11.3	97 ± 1.9	2
Pregnenolone	0.001	0.003	0.05—5	0.9995	1	93.1 ± 3.7	3.9	89.5 ± 2.7	3
					0.1	104 ± 4.3	4.2	103.5 ± 4.1	3.9

### Evaluation of CYP11A1 Activity By Quantitative Analysis of Cholesterol and Pregnenolone

**Fig. 4:**
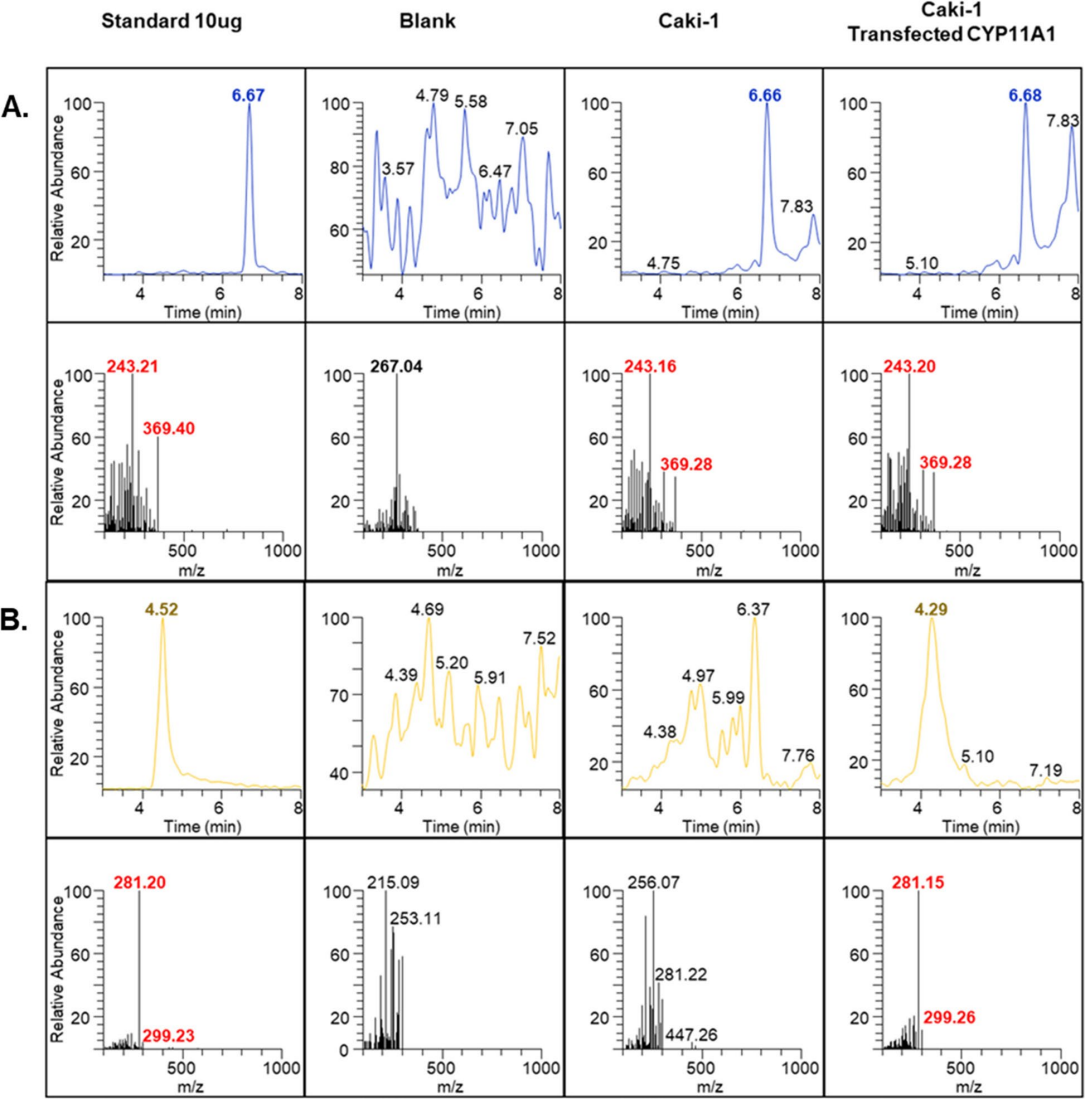
Specificity of the LC–MS/MS method established for quantitation of cholesterol and pregnenolone. Chromatographic profiles of cholesterol (**A**) and pregnenolone (**B**) in blank and treated samples. CYP11A1 activity was determined based on the levels of cholesterol and pregnenolone

The activity of CYP11A1 was determined by comparing the cholesterol and pregnenolone levels in Caki-1 cells, with and without CYP11A1 transfection. The quantitative method for steroids, with representative chromatograms and precursor ions of standard steroids, established by us, was successfully applied to determine the levels of cholesterol and pregnenolone in culture media. The specificity of the analytical method was assessed by examining the possible interference from blank medium and spiked samples after extraction. As shown in Fig. 4A, cholesterol, and pregnenolone did not interfere with one another in the matrix. As expected, cholesterol levels decreased in CYP11A1-transfected samples (Fig. 4A) whereas pregnenolone levels increased (Fig. 4B).

**Fig. 5:**
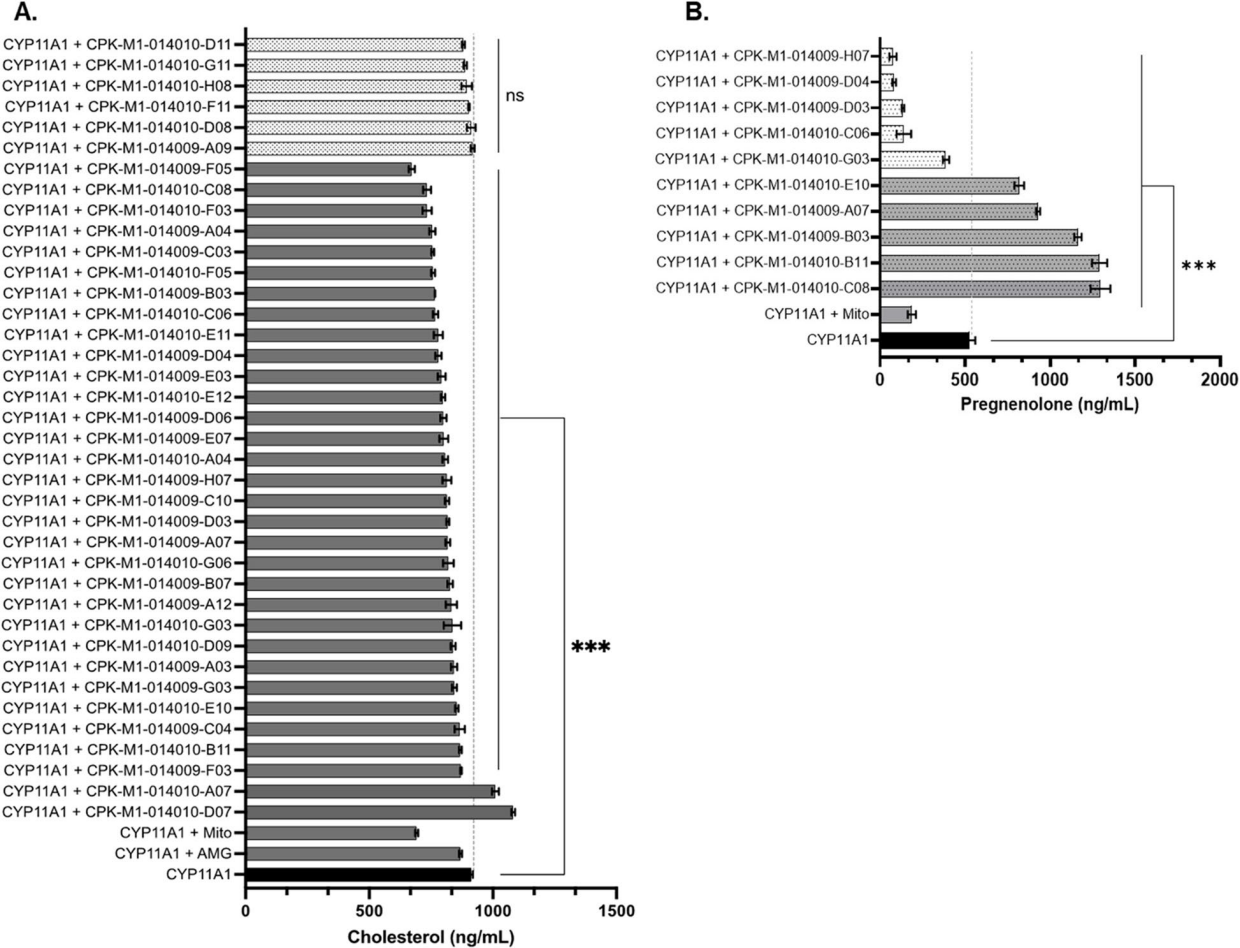
Effect of 38 selected natural compounds on the concentrations of the steroids produced. **A** Cholesterol, **B** Pregenenolone

A comparative anlaysis of the chromatographic areas revealed that 30 of the 38 compounds suppressed cholesterol levels (Fig. 5A). The major standard MS/MS fragments exhibited the same pattern as in the samples. AMG inhibited CYP11A1 activity, which was manifested as downregulation of cholesterol production. In contrast, the activation of CYP11A1 by Mito resulted in a significant decrease in cholesterol and pregnenolone levels. Most importantly, pregnenolone is the main precursor of many steroidal hormones, including progesterone, and its conversion to progesterone occurred rapidly, with most of the conversion completed during the first hour of incubation. Therefore, the reduced amount of pregnenolone after treatment with Mito could be due to its rapid conversion to progesterone. Five compounds that enhanced pregnenolone levels were detected (Fig. 5B). We also studied the mechanisms underlying the effect of the selected natural compounds. The IC_50_ for CPK-M1-014010-C08, CPK-M1-014010-B11, CPK-M1-014009-B03, CPK-M1-014009-A07, and CPK-M1-014010-E10 were 25.4, 14.6, 46.4, 26.3, and 5.9 μM, respectively. CPK-M1-014010-B1 and CPK-M1-014010-E10, which showed lower IC_50_ values, can be used as lead compounds for anticancer drugs that stimulate CYP11A1 activity. We further assessed the anticancer mechanisms of these two compounds by investigating the relevant signaling pathways affected by the them.

### Stimulation of CYP11A1 Expression and Induction of Ferroptosis Activity By the Selected Natural Compounds

**Fig. 6:**
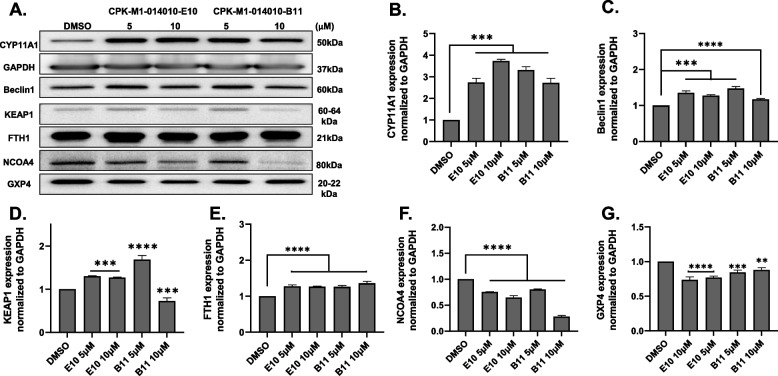
Effect of selected natural compounds on the expression of CYP11A1 and related ferroptosis markers. **A** Nontransfected Caki-1 cells were treated with two doses of natural compounds (5 and 10 µM) for 24 h and protein levels were assessed using western blotting. **B-G** Fold changes in protein levels normalized against GAPDH**p < 0.01, ***p < 0.001, and ****p < 0.0001

We further analyzed the mechanism of action of the selected natural compounds in Caki-1 cells for the following reasons: (i) these compounds significantly suppressed cell migration in the CYP11A1-overexpressing Caki-1 cell model, (ii) their low IC_50_ values indicate more potent anticancer effect, and (iii) they increased pregnenolone production while suppressing the cholesterol levels, which is indicative of the modulation of CYP11A1 activity. Among the five selected compounds that showed high efficiency in stimulating CYP11A1 activity and anticancer effects by inhibiting cell growth, two compounds (Table 3, marked with blue letters), CPK-M1-014010-E10 and CPK-M1-014010-B11, caused an increase in CYP11A1 protein levels (Fig. 6A and B). At 5 and 10 µM, these compounds increased the expression of CYP11A1 by up to threefold compared with that in the DMSO-treated group. With increasing CPK-M1-014010-E10 concentration, the CYP11A1 levels were also moderately increased, whereas at 10 µM, CPK-M1-014010-B11 showed a slight decrease in CYP11A1 levels compared with that in the 5 µM treatment. The increase in CYP11A1 levels has been reported to be associated with the increase in the levels of an autophagy marker, LC3, during the pathogenesis of preeclampsia [18]. We also observed a significant increase in the levels of another autophagy protein, Beclin1, in cancer cells, which is consistent with the upregulation of CYP11A1 expression (Fig. 6C) and expression of LC3A/B was also tested (Additional file 5). Reactive oxygen species (ROS) and lipid peroxidation products are typical hallmarks of ferroptosis [19]. We observed that CYP11A1 overexpression increased the lipid peroxidation, as did treatment with the compounds (Additional file 3). Overexpression of CYP11A1 increases ROS levels, as previously shown in our studies [14]. Moreover, excessive biosynthesis of CYP11A1 in mitochondria also leads to lipid peroxidation in BeWo cells [20]. In cellular membranes, lipid peroxidation is mainly responsible for ferroptosis, an iron-dependent cell death process [21]. We hypothesized that the remarkably impaired cancer cell proliferation could be related to CYP11A1-induced ferroptosis. To test this hypothesis, we evaluated the effect on the ferroptosis pathway by performing immunoblotting analysis of key regulators of ferroptosis markers, such as Kelch-like ECH-associated protein 1 (KEAP1), ferritin heavy chain 1 (FTH1), nuclear receptor coactivator 4 (NCOA4), and selenoprotein glutathione peroxidase 4 (GPX4) [22]. In the 5 µM treatment, KEAP1 levels were significantly increased; however, a slight decrease was observed at 10 µM, correlation to the expression of CYP11A1 was reduce in 10 µM of CPK-M1-014010-B11 treatment (Fig. 6D). Moreover, in all the compound-treated groups, FTH1 was upregulated (Fig. 6E) and NCOA4 and GPX4 were significantly downregulated (Fig. 6F and G). These results suggest that increased CYP11A1 levels strongly correspond with increases in the levels of ferroptosis-promoting proteins through ROS production and lipid peroxidation via its enzymatic activity.

## Discussion

**Fig. 7:**
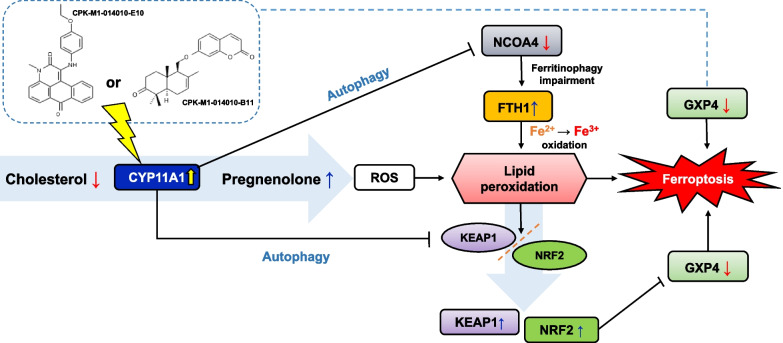
Target compounds stimulate CYP11A1 enzymatic activity and induce ferroptosis by multiple signaling pathways

Inhibition or activation of the CYP family members and an understanding of their specific involvement in cancer metabolism are important topics in anticancer drug discovery research. Here, we established a CYP11A1-overexpressing Caki-1 cell line as an efficient screening platform for the identification of active compounds against kidney cancer and used it to screen a panel of 1374 natural compounds. The activity of anticancer drug candidates was further validated for their activity on the reaction catalyzed by CYP11A1 by measuring cholesterol and pregnenolone levels using LC–MS/MS. As mentioned in the results section, CYP11A1 plays important roles in cell migration, cytotoxicity, and ferroptosis, especially in cancer cells. The implications of CYP11A1 overexpression to the ferroptosis pathway are shown in Fig. 7. These are supported by our findings that CYP11A1 induces ROS accumulation and lipid peroxidation, which are sufficient to promote ferroptosis. Li et al. [23] reported that iron-dependent lipid peroxidation regulates cell death. The depletion of NCOA4 leads to impaired ferritinophagy and unscheduled DNA synthesis [24]. Endogenous FTH1 levels are upregulated because the accumulation of cellular iron induces an increase in endogenous FTH1 [25]. The role of GPX4 inhibition has been identified in the activation of ferroptosis [26] through the transformation of glutathione to oxidized glutathione. The regulation of genes involved in oxidative stress is mostly controlled by the transcription factor nuclear factor erythroid 2 p45-related factor 2 (NRF2), which serves as the main factor in ferroptosis [27]. Normally, NRF2 and KEAP1 form a complex, and the expression of NRF2 is inhibited by its interaction with KEAP1. Under stress conditions, KEAP1 changes its conformation and disrupts the NRF2–KEAP1 interaction, resulting in the increase in NRF2 levels. This process is also regulated by the autophagy pathway, in which the cargo receptor p62/SQSTM1 competes with the NRF2–KEAP1 complex, resulting in the upregulation of NRF2. We observed the highest increase in the NRF2 levels in Caki-1 cells after CYP11A1-transfection compared with that in nontransfected control cells (Additional file 4).

In order to get insights into the transcriptional regulation of CYP11A1 with the selected compounds, molecular docking study was performed. Mitomycin C and two compounds (CPK-M1-014010-E10 and CPK-M1-014010-B11) were docked into the target protein SF1 (1YOW)- a transcriptional regulation of CYP11A1, their binding sites along with their binding affinities and the predicted binding interactions in the form of 2D diagram were shown in Additional file 6. Two selected compound were found to have binding affinities of -9.3 and -9.9 kcal/mol; whereas Mitomycin C obtained -6.9 kcal/mol. The similar amino acid interaction site of those ligands revealed interactions of two amino acids (CYS 266 and ALA 269) cloud over an aromatic group. After analyzing the binding interaction and docking scores we have found that our selected compounds have better binding energy values than the Mitomycin C in use of treatment.

Extensive evidence indicates that cholesterol can directly activate the Hedgehog signaling pathway in cancer [28]. CYP11A1 is the main enzyme catalyzing and controlling cholesterol levels in the steroidogenic pathway. The CYP11A1 activity represents an attractive therapeutic target for cholesterol-lowering medications. The activation of CYP11A1 results in a significant increase in the production of its immediate product, pregnenolone, as well as of the downstream steroids. Hsu et al. [29] demonstrated the effect of pregnenolone on the stabilization of zebrafish embryonic cell movement following CYP11A1 injection. cAMP-dependent HIPK3 action stimulates the expression of CYP11A1 by enhancement of SF-1 activity in mouse adrenocortical Y1 cells [30]. Human aldosterone synthase (CYP11B2) and cortisol synthase (CYP11B1) have been exploited in inhibitor screening campaigns to identify treatments for cardiovascular disorders [31] and cortisol-related diseases [32], which affect specific cellular responses to compounds for drug development.

In conclusion, the screening platform described here integrates the advantages of a cell-based high-throughput assay coupled with tandem mass spectrometry (LC–MS/MS) for the qualitative evaluation of enzymatic activity and allows for the flexible measurement of multiple parameters at the cellular level to facilitate more complex analyses, such as prediction of targets of carcinogenic agents. A library of 1374 compounds was investigated, which successfully revealed two selective stimulators of CYP11A1, which also show a ferroptosis activation effect. We suggest, for the first time, an association between CYP11A1 overexpression and ferroptosis and identify novel compounds that can further open up the possibility of anticancer drug development.

### Supplementary Information

**Additional file 1. **Results of wound-healing assay for 1374 compounds using CYP11A1-overexpressing Caki-1 cells. The area of the scratched portion at time 0 was set as 100%; ─ = Not determined because of detachment of cells from the surface.

**Additional file 2. **Spectra of cholesterol, pregnenolone, and internal standard finasteride. (A) Total ion chromatogram, (B) extracted ion chromatogram, (C) full-scan MS spectrum.

**Additional file 3. **Relative lipid peroxidation levels in Caki-1 cells, with or without CYP11A1 overexpression and treated with dimethyl sulfoxide (DMSO), E10 (5 and 10 μM), or B11 (5 and 10 μM).  Data are shown as fold-changes. **p < 0.01, and ****p < 0.0001.

**Additional file 4. **Enrichment of differentially expressed proteins identified using the IPA software. Important upstream signaling pathways between control and CYP11A1-overexpressing Caki-1 cells.

**Additional file 5. **Effect of selected natural compounds on the expression of CYP11A1 and related autophagy markers LC3A/B. Nontransfected Caki-1 cells were treated with two doses of natural compounds (5 and 10 µM) for 24 h and protein levels were assessed using western blotting.

**Additional file 6. **Structure-based model and docked binding with key residues in the active site of SF1 and ligands. Representative compounds (A) Mitomycin C, (B) CPK-M1-014010-E10 and (C) CPK-M1-014010-B11.

## Data Availability

Not applicable.

## Abbreviations

ACN: Acetonitrile
AMG: Aminoglutethimide
BSA: Bovine serum albumin
CYP11A1: Cholesterol side-chain cleavage enzyme
FA: Formic acid
LC–MS/MS: Liquid chromatography with tandem mass spectrometry
Mito: Mitomycin C
NPs: Natural product compounds
PBS: Phosphate-buffered saline
RCC: Renal cell carcinoma
TBS: Tris-buffered saline
TBST: TBS with 0.5% Tween 20
UHPLC: Ultra-high performance liquid chromatography
WST: Water soluble tetrazolium salt
