# Supplementary material for: Screening of Natural Compounds for CYP11A1 Stimulation Against Cell Renal Cell Carcinoma
Source: Biol Proced Online. 2023 Nov 30;25:31. doi: 10.1186/s12575-023-00225-y (PMC10687993; doi:10.1186/s12575-023-00225-y)
Supplement: Supplementary file 2 — Additional file 2. Spectra of cholesterol, pregnenolone, and internal standard finasteride. (A) Total ion chromatogram, (B) extracted ion chromatogram, (C) full-scan MS spectrum. [file 12575_2023_225_MOESM2_ESM.docx]

**
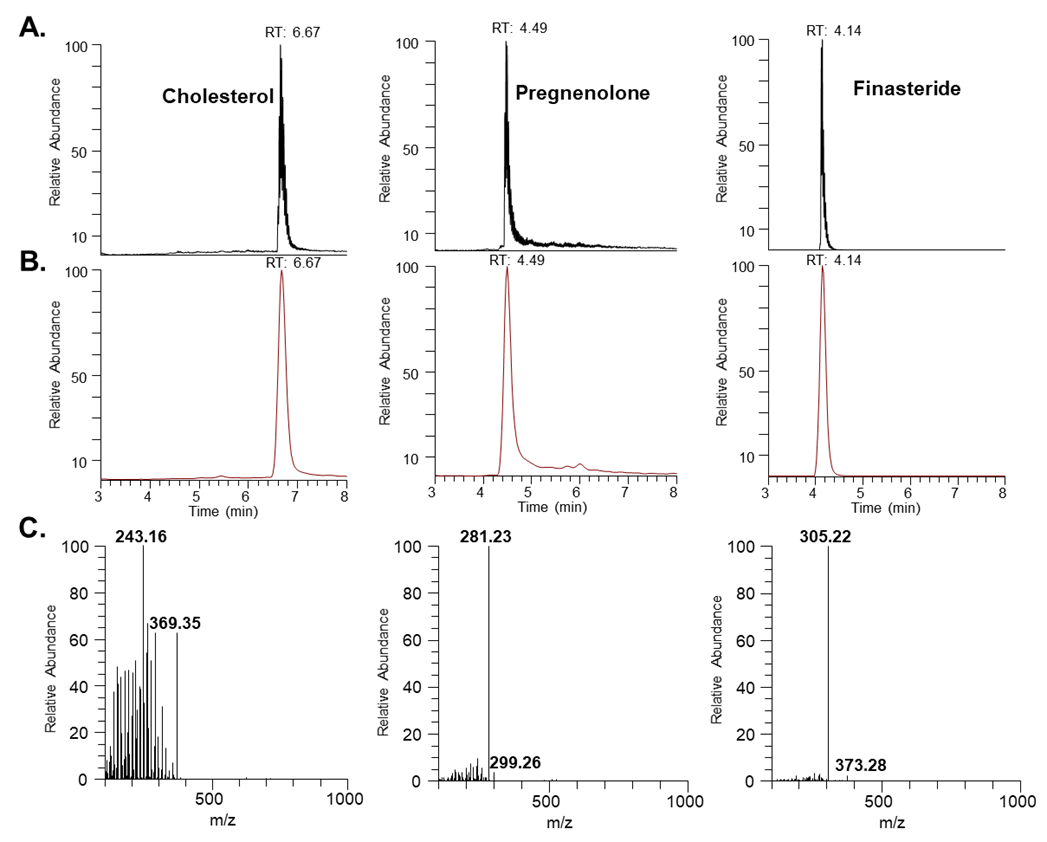
**

**Additional File 2. Spectra of cholesterol**, **pregnenolone**, **and internal standard finasteride**. **(A)** Total ion chromatogram, **(B)** extracted ion chromatogram, **(C)** full-scan MS spectrum
