# Supplementary material for: Screening of Natural Compounds for CYP11A1 Stimulation Against Cell Renal Cell Carcinoma
Source: Biol Proced Online. 2023 Nov 30;25:31. doi: 10.1186/s12575-023-00225-y (PMC10687993; doi:10.1186/s12575-023-00225-y)
Supplement: Supplementary file 3 — Additional file 3. Relative lipid peroxidation levels in Caki-1 cells, with or without CYP11A1 overexpression and treated with dimethyl sulfoxide (DMSO), E10 (5 and 10 μM), or B11 (5 and 10 μM). Data are shown as fold-changes. **p < 0.01, and ****p < 0.0001. [file 12575_2023_225_MOESM3_ESM.docx]

**
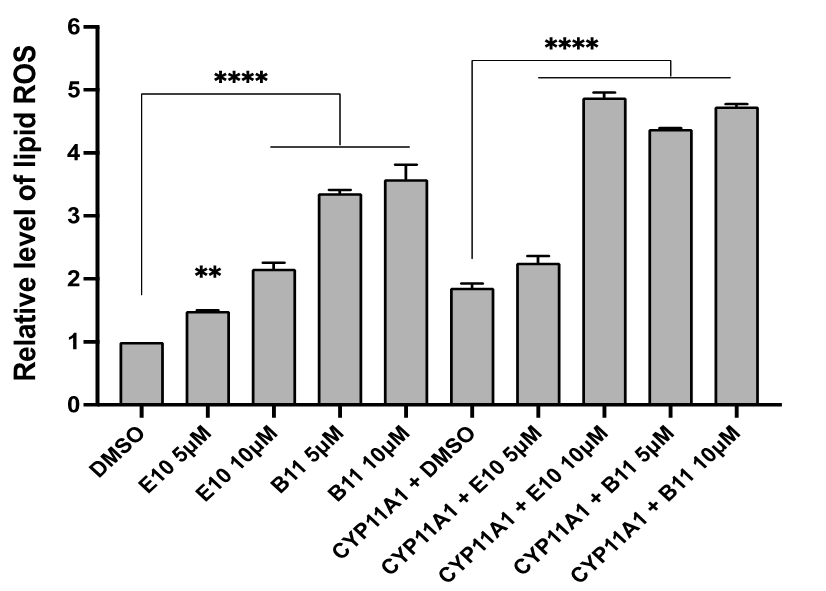
**

**Additional file 3.** Relative lipid peroxidation levels in Caki-1 cells, with or without CYP11A1 overexpression and treated with dimethyl sulfoxide (DMSO), E10 (5 and 10 μM), or B11 (5 and 10 μM).  Data are shown as fold-changes. ***p* < 0.01, and *****p* < 0.0001
