# Supplementary material for: Screening of Natural Compounds for CYP11A1 Stimulation Against Cell Renal Cell Carcinoma
Source: Biol Proced Online. 2023 Nov 30;25:31. doi: 10.1186/s12575-023-00225-y (PMC10687993; doi:10.1186/s12575-023-00225-y)
Supplement: Supplementary file 4 — Additional file 4. Enrichment of differentially expressed proteins identified using the IPA software. Important upstream signaling pathways between control and CYP11A1-overexpressing Caki-1 cells. [file 12575_2023_225_MOESM4_ESM.docx]

**
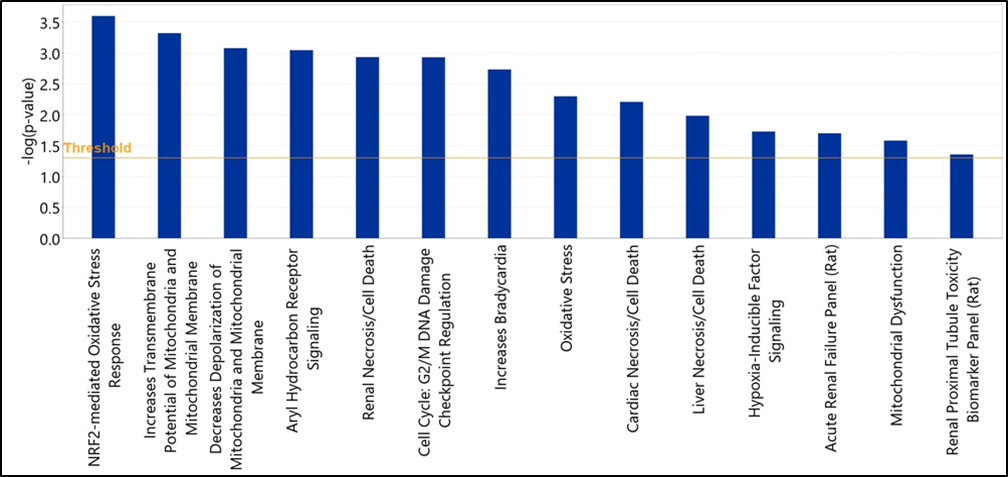
**

**Additional File 4. Enrichment of differentially expressed proteins identified using the IPA software**. Important upstream signaling pathways between control and CYP11A1-overexpressing Caki-1 cells
