# Supplementary material for: Screening of Natural Compounds for CYP11A1 Stimulation Against Cell Renal Cell Carcinoma
Source: Biol Proced Online. 2023 Nov 30;25:31. doi: 10.1186/s12575-023-00225-y (PMC10687993; doi:10.1186/s12575-023-00225-y)

**Additional File 5. Effect of selected natural compounds on the expression of CYP11A1 and related autophagy markers LC3A/B.** Nontransfected Caki-1 cells were treated with two doses of natural compounds (5 and 10 µM) for 24 h and protein levels were assessed using western blotting.


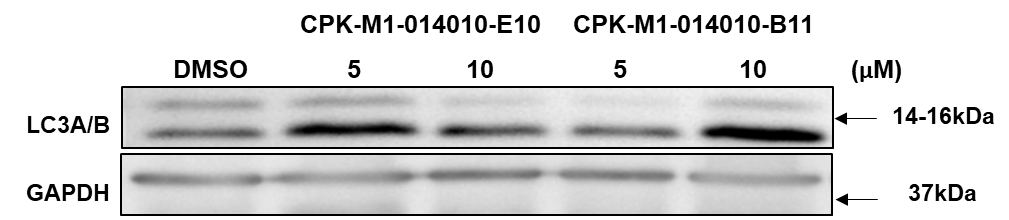

Supplement: Supplementary file 5 — Additional file 5. Effect of selected natural compounds on the expression of CYP11A1 and related autophagy markers LC3A/B. Nontransfected Caki-1 cells were treated with two doses of natural compounds (5 and 10 µM) for 24 h and protein levels were assessed using western blotting. [file 12575_2023_225_MOESM5_ESM.docx]
