# Supplementary material for: Screening of Natural Compounds for CYP11A1 Stimulation Against Cell Renal Cell Carcinoma
Source: Biol Proced Online. 2023 Nov 30;25:31. doi: 10.1186/s12575-023-00225-y (PMC10687993; doi:10.1186/s12575-023-00225-y)

**Additional File 6.** Structure-based model and docked binding with key residues in the active site of SF1 and ligands. Representative compounds (A) Mitomycin C, (B) CPK-M1-014010-E10 and (C) CPK-M1-014010-B11.


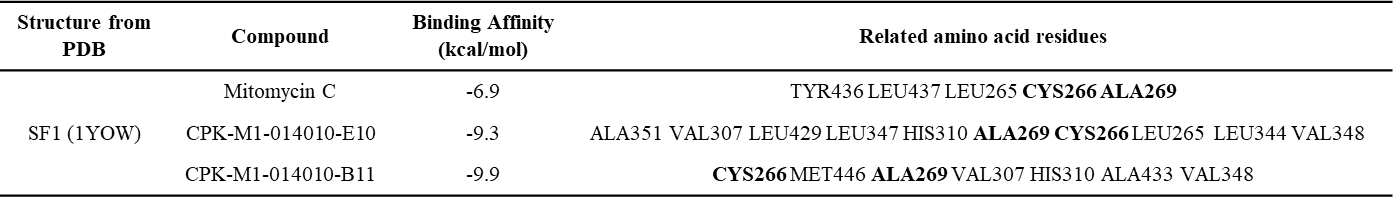


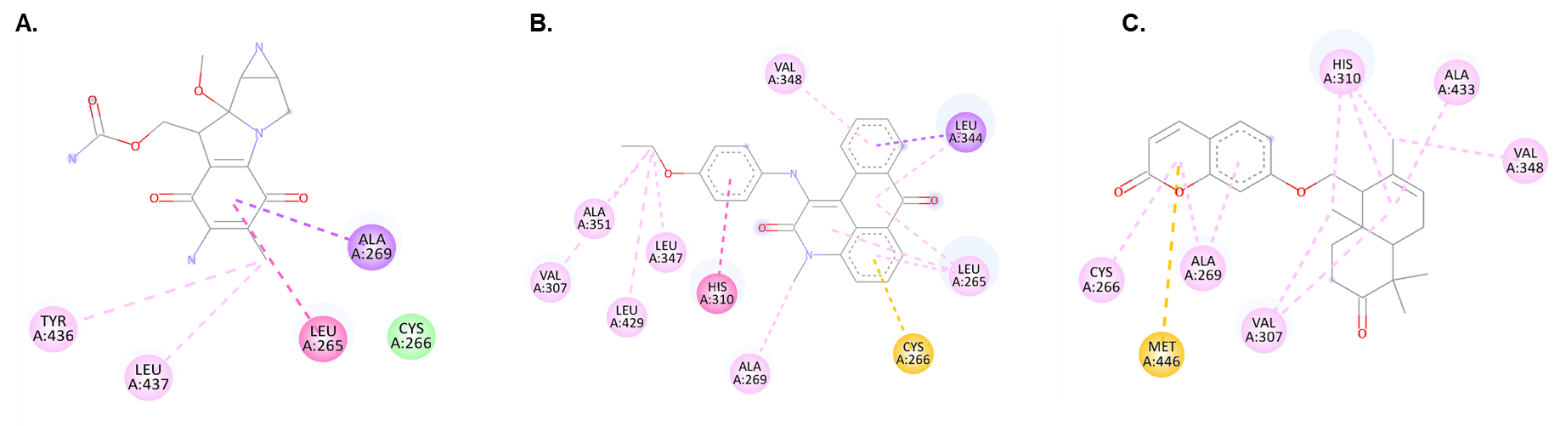

Supplement: Supplementary file 6 — Additional file 6. Structure-based model and docked binding with key residues in the active site of SF1 and ligands. Representative compounds (A) Mitomycin C, (B) CPK-M1-014010-E10 and (C) CPK-M1-014010-B11. [file 12575_2023_225_MOESM6_ESM.docx]
